# Probiotic-derived heptelidic acid exerts antitumor effects on extraintestinal melanoma through glyceraldehyde-3-phosphate dehydrogenase activity control

**DOI:** 10.1186/s12866-022-02530-0

**Published:** 2022-04-22

**Authors:** Shotaro Isozaki, Hiroaki Konishi, Hiroki Tanaka, Chikage Yamamura, Kentaro Moriichi, Naoki Ogawa, Mikihiro Fujiya

**Affiliations:** 1 Division of Metabolism and Biosystemic Science, Gastroenterology, and Hematology/Oncology, Department of Medicine, Asahikawa Medical University, Asahikawa, 078-8510 Japan; 2 Department of Forensic Medicine, Tokai University School of Medicine, Isehara, 259-1193 Japan; 3 Department of Gastroenterology and Advanced Medical Sciences, Department of Medicine, Asahikawa Medical University, Asahikawa, 078-8510 Japan; 4 Division of Tumor Pathology, Department of Pathology, Asahikawa Medical University, Asahikawa, 078-8510 Japan; 5 Center for Advanced Research and Education, Asahikawa Medical University, Asahikawa, 078-8510 Japan

**Keywords:** Glyceraldehyde-3-phosphate dehydrogenase, Heptelidic acid, Melanoma

## Abstract

**Background:**

Several microorganisms inhabit the mammalian gastrointestinal tract and are associated with the pathogenesis of various diseases, including cancer. Recent studies have indicated that several probiotics produce antitumor molecules and inhibit host tumor progression. We demonstrated that heptelidic acid (HA), a sesquiterpene lactone derived from the probiotic *Aspergillus oryzae*, exerts antitumor effects against pancreatic cancer in vitro and in vivo. In this study, the antitumor effects of HA against extraintestinal melanoma were assessed in vitro and in vivo.

**Results:**

Sulforhodamine B (SRB) assay revealed that the growth of B16F10 cells was significantly inhibited by HA in a concentration-dependent manner. The enzymatic activity of glyceraldehyde-3-phosphate dehydrogenase (GAPDH) decreased in proportion with the growth inhibition effect of HA. Moreover, oral HA administration significantly suppressed the growth of transplanted B16F10 tumors without any significant changes in biochemical test values. Moreover, GAPDH activity in the transplanted tumor tissues in the HA group significantly decreased compared with that in the PBS group.

**Conclusion:**

This study suggests that orally administered HA was absorbed in the gastrointestinal tract, reached the cancer cells transplanted in the skin, and inhibited GAPDH activity, thereby inhibiting the growth of extraintestinal melanoma cells. Thus, this study proposes a novel system for extraintestinal tumor regulation via gut bacteria-derived bioactive mediators.

**Supplementary Information:**

The online version contains supplementary material available at 10.1186/s12866-022-02530-0.

## Background

Several microorganisms inhabit the gastrointestinal tract and have a symbiotic relationship with their mammalian hosts. Previous studies have demonstrated that the disruption of intestinal microflora is closely associated with the pathogenesis of various diseases, such as atopic dermatitis, inflammatory bowel disease, and cancer [1–3]. Several studies have indicated that oral probiotic administration and fecal microbiota transplantation (FMT) are potentially effective methods to prevent and treat such diseases [4–6].

Recently, some bacterial molecules produced by probiotics have been identified to possess antitumor properties. Tsai et al. revealed that antimicrobial peptides such as m2163 and m2386 induced colorectal cancer cell apoptosis in vitro [7]. Hatakeyama et al. determined that a Natto peptide can reduce the number of uterine and cervical cancer cells in vitro [8]. Ferrichrome derived from *Lactobacillus casei* also induced the apoptosis in gastrointestinal cancer cells, including colorectal, gastric, and pancreatic cancer cells [9–12]. These studies suggest that bacterial molecules directly inhibit tumor cell progression, thus providing health benefits to mammalian hosts.

*Aspergillus oryzae* is an imperfect fungus that is used in the production of Japanese fermented food, including soybean paste and soy sauce, since ancient times. Recent studies have presented the beneficial effects of foods fermented by *A. oryzae*, e.g., conventional intake of soybean paste fermented by *A. oryzae* have a preventive effect on lifestyle-related diseases such as hypertension and type II diabetes [13–15]. Interestingly, fermented soybean paste also shows antitumor activity against various cancers, including extraintestinal tumors [16–18]. Recently, we demonstrated that heptelidic acid (HA) from *A. oryzae* shows antitumor effects against pancreatic cancer cells [19]. In this previous study, a bacterial culture supernatant was collected and dissociated using HPLC, and HA was identified as an antitumor mediator derived from *A. oryzae*. We also performed an ex vivo study on mice intestinal loop and determined that HA in a resected mouse intestine passes through the intestinal wall and inhibits the progression on pancreatic cancer cells. This indicates that some probiotic molecules are absorbed by the host, circulate in the whole body, and directly affect digestive lesions. However, it has not been clearly understood whether such probiotic molecules exert antitumor effects on tumors other than those in the digestive tract when administered in the gastrointestinal tract in vivo.

HA was first identified from *Trichoderma koningii* as a specific and irreversible inhibitor of glyceraldehyde 3-phosphate dehydrogenase (GAPDH) [20]. GAPDH is an important enzyme involved in cellular metabolism. In the glycolytic/gluconeogenic metabolic pathways, GAPDH converts glyceraldehyde 3-phosphate to 1,3-bisphosphoglycerate using NAD(P)^+^ as a coenzyme. Moreover, numerous moonlighting activities of GAPDH have been reported in the literature. Majority of these activities are irrelevant to its main function in energy metabolism; it is involved in apoptotic–autophagic cell death, DNA repair, tRNA export, as well as membrane fusion and transport [21–24]. GADPH was also highlighted as a therapeutic target for inhibiting the abnormal tumor glycolysis pathway called the Warburg effect in malignant tumors [25, 26]. However, whether orally administered HA affects the GAPDH activity of extraintestinal tumors, including melanoma, and exhibits antitumor functions has not been elucidated.

This study assessed the growth and GAPDH inhibitory effects of orally administered HA on extraintestinal melanoma using in vitro cell proliferation assay and in vivo homograft melanoma mouse model.

## Methods

### Materials

B16F10 cells were obtained from Dr. Takayuki Ohkuri (Department of Pathology, Asahikawa Medical University, Asahikawa, Japan.). High-glucose Dulbecco’s modified Eagle’s medium was purchased from FUJIFILM Wako Pure Chemical, Osaka, Japan. Fetal bovine serum, L-glutamine, penicillin, and streptomycin were purchased from Thermo Fisher Scientific, MA, USA. Sulforhodamine B was purchased from Merck KGaA, Darmstadt, Germany. Heptelidic acid was purchased from Adipogen Life Sciences, CA, USA. A GAPDH activity assay kit was purchased from Abcam, Cambridge, UK.

### Cell culture

B16F10 cells are murine melanoma cells that originated from a C57BL/6 J mouse. These cells were cultured in a high-glucose Dulbecco’s modified Eagle’s medium (FUJIFILM Wako Pure Chemical) supplemented with 10% (vol/vol) fetal bovine serum, 2 mM L-glutamine, 50 U/mL penicillin, and 50 mg/mL streptomycin (Thermo Fisher Scientific, MA, USA).

### Sulforhodamine B assay

B16F10 cells were seeded onto 96-well microplates at 1 × 10^4^ cells/well and cultured for 24 h. After 24 h of HA treatment (*n* = 5) at different concentrations (0, 10, 10^2^, 10^3^, and 10^4^ ng/mL), the cells were fixed in 10% trichloroacetic acid for 1 h at 4 °C and washed four times in distilled water. The microplates were then dehydrated at room temperature, stained with 0.057% (wt/vol) Sulforhodamine B (SRB) in 1% (vol/vol) acetic acid at 100 μL per well, washed four times with 0.1% acetic acid, and re-dehydrated at room temperature. The stained cells were lysed in 10 mM unbuffered Tris base solution, and the optical density was measured at 510 nm [27].

### Cell migratsion assay

B16F10 cells were seeded on 12-well microplates at 1 × 10^5^ cells per well and cultured for 24 h. Scratches were made using a sterile 200-μL pipette tip; subsequently, HA was added to the HA group (final concentration of HA: 1 μg/mL) and the cells were cultured for 24 h (*n* = 3). The areas of the scratches were recorded using a digital camera, and the distance of scratches was measured using an ImageJ software program [28].

### GAPDH activity assay

The activity of GAPDH was determined using a GAPDH Activity Assay Kit (Abcam) following the manufacturer’s instructions. B16F10 cells were seeded onto 12-well microplates at 1 × 10^5^ cells/well and cultured for 24 h. After 24 h of HA treatment (*n* = 3) at different HA concentrations (0.025, 0.05, 0.25, 0.5, and 2.5 μg/mL), the cells were lysed using GAPDH assay buffer and the its activity was determined (mU/mL). The GAPDH activity in tumors from B16F10 graft mice model was also determined following the manufacturer’s protocol.

### Study animals

This study was approved by the Institutional Animal Care and Use Committee of the Asahikawa Medical University (Approval number: R3-113). C57BL/6 mice were purchased from Charles River Laboratories Japan Inc. (Yokohama, Japan).

### Homografts

B16F10 cells (2 × 10^6^ cells/35 μL phosphate buffer saline [PBS]/tumor) were mixed with Matrigel (15 μL/tumor). After removing all hair, 50 μL of cell suspension was subcutaneously injected into the backs of the 6-week-old mice. The mice were then randomly divided into groups based on whether they received phosphate buffer saline (PBS) or HA, with five mice per group. Subsequently, 100 μL of PBS or 10 μg/100 μL PBS of HA was orally administered daily, starting with the day after the injection of B16F10 cells. The tumor size was calculated using the following formula: tumor size (mm^2^) = (major diameter) × (minor diameter). Whole blood was collected from the inferior vena cava and subjected to centrifugation at 2,500 × g for 10 min at room temperature, and the serum was obtained for mice in both groups. The serum samples were stored at − 80 °C and sent for biochemical examination to Oriental Yeast Co., Ltd. (Tokyo, Japan).

### Statistical analysis

The assay data were analyzed using Student’s unpaired t-test. A *p*-value of < 0.05 was considered statistically significant.

## Results

### HA exhibited cytostatic activity and GAPDH inhibition in melanoma-derived B16F10 cells

**Fig. 1:**
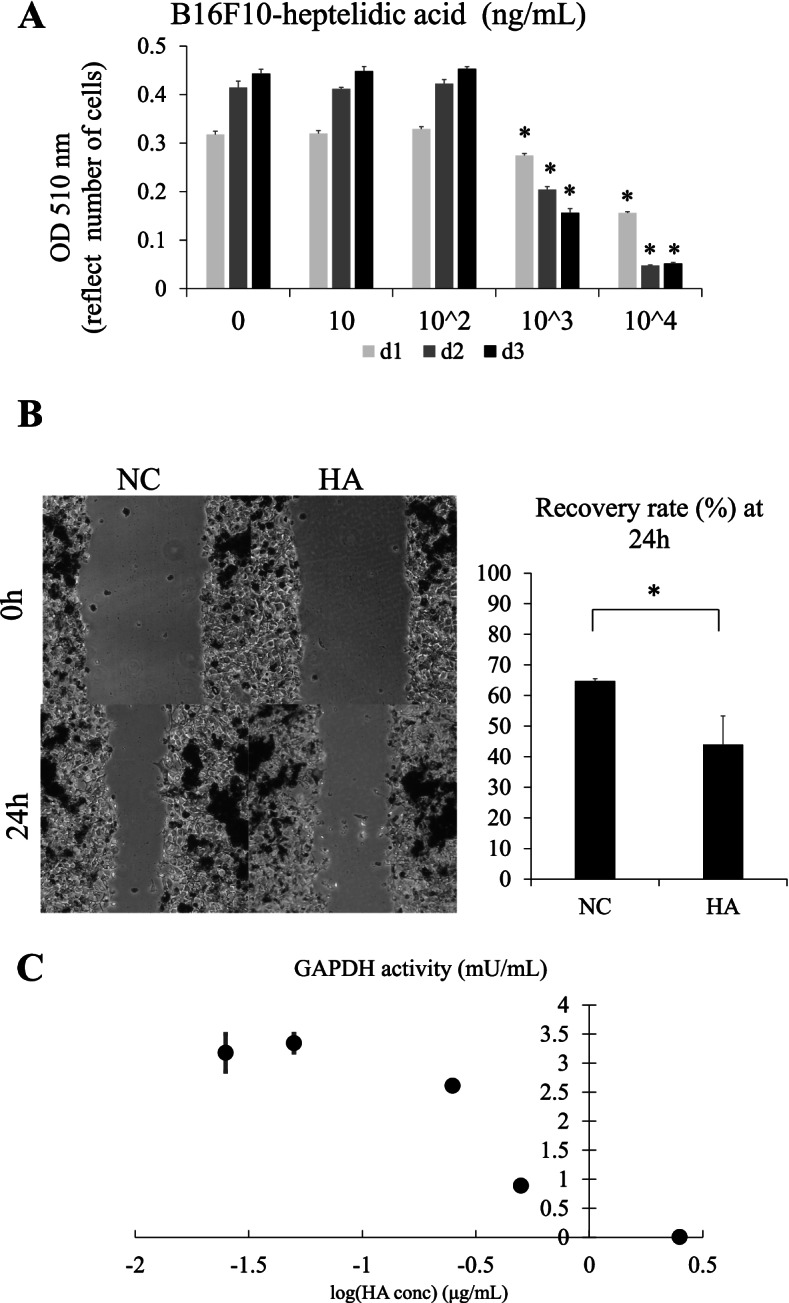
Heptelidic acid (HA) exerts an antiproliferative effect via inhibiting GAPDH in vitro. A sulforhodamine B assay revealed that HA exerted growth-suppressing effects on B16F10 cells in a concentration-dependent manner (**A**). A cell scratch assay indicted that HA inhibited the migration of B16F10 cells (**B**). GAPDH activity assay revealed that HA inhibited GAPDH activation in a concentration-dependent manner (**C**). The error bars represent the standard deviation (SD). * *p* < 0.05 by Student’s t-test

To assess whether HA exerts an antiproliferative effect on melanoma-derived B16F10 cells, an SRB assay was performed. The growth of B16F10 cells was significantly suppressed by HA in a concentration-dependent manner on Days 2 and 3 (Fig. 1A). To investigate whether HA suppresses the migration of melanoma cells, a cell-scratch assay was performed. Cell migration significantly reduced after treatment with 1 μg/mL HA for 24 h (Fig. 1B), indicating that HA exerts an antiproliferative effect by suppressing the cell growth and migration of melanoma cells.

### HA inhibits GAPDH activity in melanoma cells

Previous studies have suggested that HA irreversibly inhibits GAPDH activity, thereby exerting a growth-suppressive effect, particularly on cancer cells that depend on the Warburg effect [29]. Therefore, GAPDH activity in HA melanoma cells was assessed, and it was observed that HA inhibited the GAPDH activity in a concentration-dependent manner (Fig. 1C). Notably, the reduction in GAPDH enzymatic activity and cell density was parallelly shifted, indicating that the growth-suppressive function of HA was mediated by the inhibition of GAPDH activity in melanoma cells.

### Orally administered HA exerted an antiproliferative effect on in vivo B16F10 homograft mice model

**Fig. 2:**
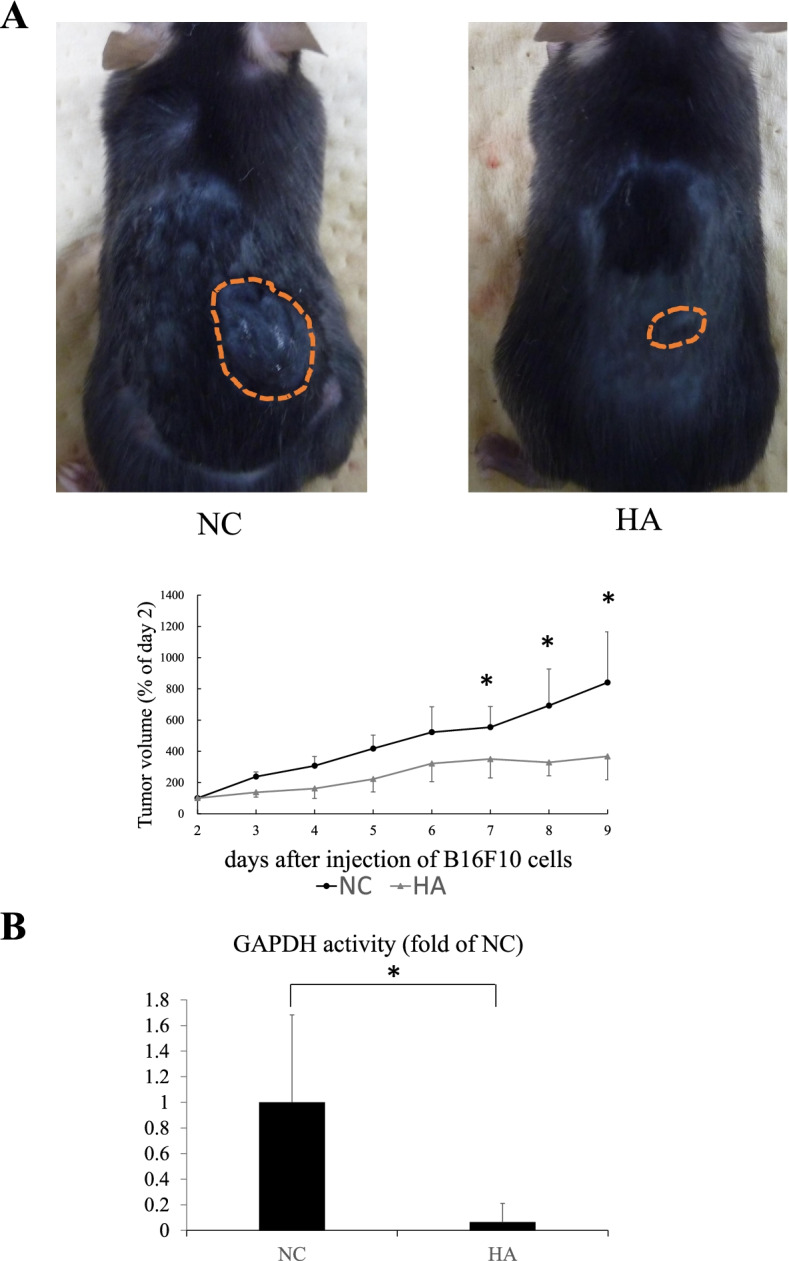
Heptelidic acid (HA) inhibited the tumor growth in an in vivo homograft model. B16F10 cells were transplanted into C57BL/6 mice and 10 μg of HA was orally administered daily. Tumor growth was significantly inhibited after HA treatment (**A**). GAPDH activity assay showed that the tumor GAPDH activity in the HA group was significantly decreased compared with that in the negative control group (**B**). The error bars represent the standard deviation (SD). * *p* < 0.05 by Student’s t-test

To confirm whether HA exerts tumor-suppressive effects in vivo, B16F10 cells were transplanted into C57BL/6 mice and 10 μg of HA was orally administered daily. The tumor size significantly decreased after HA administration (Fig. 2A).

### HA inhibited GAPDH activity in the grafted tumor

GAPDH activity assay was performed using isolated tumors to assess whether the activity in the transplanted tumor cells decreased after HA administration. The tumor GAPDH activity in the HA group significantly decreased by 0.07 times compared with that in the PBS group (Fig. 2B), suggesting that orally administered HA was absorbed through the intestinal lumen, reached the location of the transplanted tumor, and inhibited the GAPDH activity, thereby exerting antitumor effects.

### HA exerted tumor-suppressive effects with less adverse effects

**Fig. 3:**
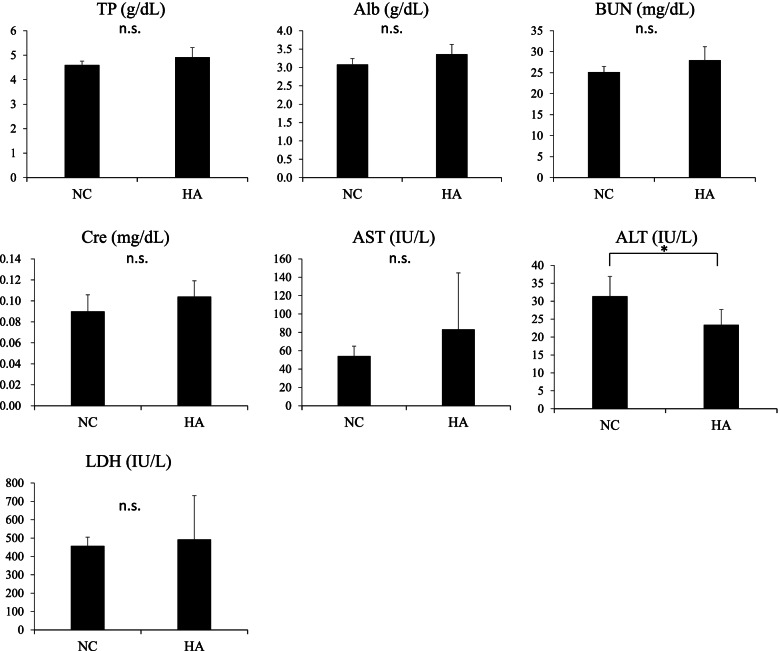
Heptelidic acid (HA) treatment did not affect the results of serum biochemistry tests. Biochemical analysis showed no significant differences between the HA and negative control group in terms of TP, Alb, BUN, Cre, AST, and LDH values. The ALT level was significantly decreased after HA treatment. The error bars represent the standard deviation (SD). * *p* < 0.05 by Student’s t-test. TP, total protein; Alb, albumin; BUN, blood urea nitrogen; Cre, creatinine; AST, aspartate aminotransferase; ALT, alanine aminotransferase; LDH, lactate dehydrogenase

To assess whether HA induced tissue damage in vivo, biochemical testing was performed, the results of which revealed that the values of alanine transaminase in the HA group were significantly lower than those in the PBS group. The levels of total protein, albumin, blood urea nitrogen, creatinine, aspartate aminotransferase, and lactate dehydrogenase were not significantly different in the HA and PBS groups (Fig. 3).

## Discussion

This study determined that HA derived from *A. oryzae* exerts a strong tumor-suppressive effect on melanoma-derived B16F10 cells in an in vitro and in vivo homograft model. Oral HA treatment suppressed the growth of the extraintestinal tumors that were grafted under the skin of the back. A GAPDH activity assay indicated that HA treatment significantly decreased the GAPDH activity in directly treated B1610 cells in vitro and orally treated in vivo homograft mouse models. Therefore, HA may be one of the key molecules of the probiotic *A. oryzae* that inhibit extraintestinal tumor growth. This study proposes a novel extraintestinal tumor regulation system using bacteria-derived bioactive mediators.

In the past several decades, numerous investigations have demonstrated the efficacy of probiotics in the treatment of gastrointestinal cancer in preclinical tests. For instance, several *Lactobacillus* strains inhibited cell proliferation by inducing cell-cycle arrest in vitro [30] and inhibited the growth of colon cancer in dimethyl hydrazine-induced Sprague Dawley rats by inducing apoptosis in vivo [31]. Interestingly, probiotics and foods fermented by probiotics have therapeutic effects on various cancer types in vitro and in vivo. For instance, Kaga et al. showed that breast cancer progression is inhibited by the administration of *L. casei* Shirota in a rat chemical carcinogenesis model [32]. Fatahi et al. revealed that probiotic-fermented kefir exhibits antitumor activity against glioblastoma cells in vitro [33]. Greathouse et al. indicated that replenishing *Lactobacillus* and *Bacteroides* in the gut, which were decreased due to chemotherapy, can improve the efficacy of therapies for lung cancer [34]. Another clinical study revealed that FMT from a patient with melanoma who responded to immune checkpoint inhibitor (ICI) therapy improved the therapeutic response of an ICI-refractory patient by altering the intestinal flora [6]. These results indicate that probiotics and intestinal microbes can modulate the homeostasis of extraintestinal organs. However, the underlying mechanism of how microbiome affects the extraintestinal tumor progression and its mediators was poorly understood.

Bioactive natural products from microorganisms have been investigated to understand the symbiotic relationship between microbes and mammalian hosts. Some bacterial molecules, including lipopolysaccharides and flagellin, stimulate the toll-like receptor pathway in host epithelial and immune cells and are associated with the maintenance of host homeostasis. Septic shock and multiple organ failure occur when these molecules infiltrate the host’s body under conditions such as intestinal disorders [35, 36]. In this study, we demonstrated that HA, a probiotic molecule derived from *A. oryzae*, passes through the gastrointestinal tract and reaches the extragastrointestinal malignancy without losing its activity and suppresses tumor progression. This suggests that mammals have unique systems that would benefit them by mediating the function of bacterial molecules under an appropriate organic environment.

Previously, it was demonstrated that HA passes through the intestinal tract and exerts its antiproliferative effects on pancreatic cancer cells in an ex vivo study on mice intestinal loop [19]. However, whether orally administered HA reaches extra-gastrointestinal tumors was not clarified in that study. In the present study, the GAPDH inhibitory effect, which were assumed to be due to HA, was confirmed in transplanted tumors by HA administration in vivo, as seen in Fig. 2B. This strongly suggests that orally administered HA passes through the gut, is circulated in the bloodstream, reaches the transplanted tumors, and inhibits tumor growth in vivo.

The Warburg effect was first reported by Otto Warburg as a characteristic of glucose metabolization in cancer cells [37]. This report stated that glycolysis is activated even in the presence of sufficient oxygen and produces large amounts of lactate, thereby supporting tumor cell progression. Over the past decades, compounds that inhibit the Warburg effect were identified and their potential in cancer therapeutics was tested in in vitro clinical studies [38–40]. GAPDH is a rate-determining enzyme associated with the Warburg effect and it converts glyceraldehyde 3-phosphate (G3P) to D-1,3-bisphosphoglycerate (1,3-BPG). Tumor cells are highly dependent on the GAPDH-mediated glycolysis pathway for ATP production [29]. In this study, the in vitro and in vivo HA treatments downregulated GAPDH activity in melanoma cells, suggesting that the extraintestinal tumor-suppressive effects of HA were mediated by GAPDH inactivation. Regarding the observed adverse events, HA did not result in abnormal biochemical test results in vivo, indicating the little effect of the effective dose of HA as part of cancer therapy on hepatic and renal functions. Meanwhile, a high HA dose was reported to suppress the growth of non-cancerous cells, such as endothelial cells and fibroblasts, in vitro [41]. This might suggest that HA has a high affinity to tumor-associated antigens compared with non-cancerous cells; thus, an appropriate HA dose has a therapeutic effect without cytotoxicity.

## Conclusion

We demonstrated that HA derived from *A. oryzae* HA exerts antiproliferative effects by inhibiting GAPDH in melanoma-derived B16F10 cells. Furthermore, orally administered HA exerted antitumor effects on subcutaneously grafted tumor cells via GAPDH inhibition in vivo, indicating that novel interactions occur between mutualistic　bacteria and host tumors located in the distant gastrointestinal tract.

## Supplementary Information

**Additional file 1: Supplementary Table 1.**

## Data Availability

All data generated or analyzed in this study are included in this published article and its supplementary Table 1.
